# Long-term host parasite dynamics in eight odontocete species from south-eastern South Africa

**DOI:** 10.1016/j.ijppaw.2025.101133

**Published:** 2025-09-05

**Authors:** Inge A. Adams, Natasha Roussouw, Cecile Reed, Gin Swen Ham, Stephanie Plön

**Affiliations:** a Department of Biological Sciences, University of Cape Town, South Africa; b BioConsult SH, Husum, Germany; c Two Oceans Aquarium, Cape Town, South Africa; d School of Life and Health Sciences, University of Roehampton, London, United Kingdom

**Keywords:** Parasites, Odontocetes, Delphinids, South Africa, Strandings, Bycatch, Health

## Abstract

Although investigations of stranded and bycaught cetaceans have been conducted since the 1970s, little is known about parasites infecting the 25 species found in the South African subregion. For this study, we retrospectively identified parasites from eight bycaught and stranded odontocete species along the south-eastern coast of South Africa between 1970 and 2015 to produce a list of parasite species affecting southern African odontocetes and examine patterns of infection in host species with regards to age, sex, time intervals and collection method. Parasites were identified in 192 out of the 2599 individuals examined. Previously unreported parasite species were found for several southern African odontocete species, underscoring the importance of museum collections for biological research. Offshore and stranded odontocetes generally had a higher prevalence of parasites, although there were a few exceptions. Binomial logistic regression models showed significantly higher probability of parasitic presence (*p* < 0.05) in stranded *Delphinus delphis* than bycaught individuals, and significantly lower probability of parasitic presence (*p* < 0.05) in neonates and calves of stranded *Tursiops aduncus* and *Stenella coeruleoalba* than adult animals. No significant difference (*p* > 0.05) was detected between the sexes of all odontocete species investigated (*p* > 0.05). Parasitic prevalence was highest during the most recent years (2000–2015) for half of the odontocete species investigated, in agreement with the models. Accurate morphological identification of parasites affecting marine mammals is essential for comprehending disease epidemiology, assessing the health of cetacean populations, and developing effective conservation and management strategies.

## Highlights

• Examined parasites collected between 1970 and 2015 off south-eastern South Africa.
• Parasites originated from bycaught and stranded individuals from 8 odontocete species.
• Study identified parasites not previously found in southern African odontocetes.
• Found significant differences in parasite presence for certain odontocete species.
• Study highlights importance of long-term data and use of museum collections.

## Graphical abstract

**Figure:**
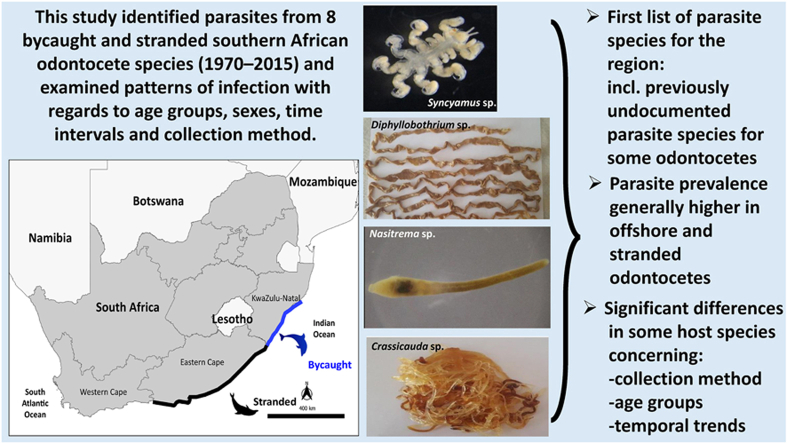

## 1 Introduction

Marine mammals, including cetaceans, are considered good indicators of the health of marine environments, because they have long life spans, occupy the highest trophic level in the food chain, and bio-accumulate toxins present in the marine environment (Fair and Becker, 2000; Aznar-Alemany et al., 2019; Plön et al., 2024). In addition, they can act as good sentinels for human health as they consume many of the same fish species caught for human consumption and share similar life history traits, such as long-life spans, late maturity, low reproductive output, and high trophic level (Fair and Becker, 2000). Eight cetacean species found in the South African sub-region, which can function as indicators of Ocean Health, include the pygmy sperm whale (*Kogia breviceps*), the dwarf sperm whale (*Kogia sima*), the striped dolphin (*Stenella coeruleoalba*), the pantropical spotted dolphin (*Stenella attenuata*), the Risso's dolphin (*Grampus griseus*), the common dolphin (*Delphinus delphis*), the Indo-Pacific bottlenose dolphin (*Tursiops aduncus*), and the Indian Ocean humpback dolphin (*Sousa plumbea*).

The latter three species occupy mostly coastal and/or inshore habitats (Best, 2007; Plön et al., 2012), which are greatly affected by human activities and usually display significant environmental degradation as has been shown to affect other cetaceans along the South African coastline (Lane et al., 2014; Gui et al., 2016; Aznar-Alemany et al., 2019; Plön et al., 2023). This may result in immune system suppression, making cetaceans more vulnerable to diseases and parasitic infection (Reckendorf et al., 2023). A review of the population structure of *T. aduncus* as part of the South African Red List assessment (Cockcroft et al., 2016) recognised three subpopulations along the South African coast being listed as “vulnerable”, “near threatened” and “data deficient”, respectively. Additionally, the International Union for the Conservation of Nature (IUCN; Braulik et al., 2015, 2023) and the Red List of Mammals of South Africa (Plön et al., 2016) listed *S. plumbea* as being “endangered”; the only resident marine mammal species in South African waters with this classification at present. Despite the high conservation priority of these species, research investigating parasitic infections in South African coastal dolphins, which can be an indicator of population health, is limited.

Understanding the role parasites play in animal and ecosystem health is important. While parasites have been shown to be possible indicators of the health of ecosystems (Tramboo et al., 2022), they can also negatively affect marine mammal and human health. For instance, various diseases are caused by parasites, which can have a significant effect on marine mammal populations (Harwood and Hall, 1990; Dubey et al., 2003), even causing death in some cases (e.g., Cuvertoret-Sanz et al., 2020). Exposure to certain pollutants, such as persistent organic pollutants (POPs), can adversely affect the immune system of cetaceans, rendering them more vulnerable to parasitic infections. However, this relationship is complex, with parasites possibly influencing the host's capacity to metabolize pollutants, and *vice versa* (Gui et al., 2018). Certain parasite species (e.g., *Anisakis* sp. or *Bolbosoma* sp.) also have the potential to infect humans through the ingestion of infected undercooked fish leading to gastrointestinal issues (Arizono et al., 2012; Rahmati et al., 2020). Thus, parasitic assemblage data for host species is valuable as it can provide important information on host diet, population structure and species movement, which is vital for population conservation and management (Aznar et al., 1995; MacKenzie, 2002; Cantatore and Timi, 2015). A number of factors influence the way the host is affected by parasitic infections. These include the specific type of parasite infecting the individual host, abundance of both a certain parasite and various parasites present in the individual host, and the overall health of the individual host (i.e. whether the host is already immunocompromised or not; Duignan, 2003). For example, a certain type of parasite can infect the digestive tract of host species leading to ulcer formation, internal bleeding and blockages, which can cause the host to become malnourished (Dailey, 2001). In addition, parasitic infection can lead to a stranding event, especially if the host species is already immunocompromised and parasites manage to infect the brain, ears, sinuses, or nervous system (Duignan, 2003). Other factors that could affect the intensity of parasite infection include age class and sex of the host species. For example, in Australia, the barnacle *Xenobalanus globicipitis* was found to be more prevalent on younger *Tursiops truncatus* individuals when compared to mature individuals (Orams and Schuetze, 1998). While research on cetaceans is ongoing, investigations involving other animal species, including birds, rodents, ungulates and humans, illustrate how differences in parasite infections based on sex can emerge as a result of variations in immune responses, social behaviours, and habitat utilization (Wesołowska, 2022).

The first systematic health investigations of dolphins in the Southern Hemisphere were conducted by Lane et al. (2014) on incidentally bycaught dolphins from the bather protection nets (BPNs) off KwaZulu-Natal, South Africa. Their research suggested a rise in the occurrence of parasitic lesions among these dolphins, providing important baseline data on conditions affecting coastal dolphins in southern Africa. Another study by Van Bressem et al. (2020) investigated the prevalence of cranial crassicaudiasis in *T. aduncus* and *S. plumbea* from the same region. The prevalence rates observed in *S. plumbea* and *T. aduncus* were 13 % and 31.9 %, respectively, with the study suggesting that *Crassicauda* spp. could contribute to the natural mortality rates in these two dolphin species (Van Bressem et al., 2020). Knowledge on the parasites infecting these odontocetes is thus important as is the investigation of any trends in infection to be able to inform future health studies.

The continued bycatch of dolphins in the bather protection nets (BPN) along the KwaZulu-Natal coastline, South Africa, has led to detailed investigations to determine the general health status, natural history, and trophic ecology of the species (Plön et al., 2015). BPNs are managed and maintained by the KwaZulu-Natal Sharks Board (KZNSB) to reduce the risk of shark-human interactions (Dudley, 1997; Cliff and Dudley, 2011). It is assumed that animals get caught in nets accidently and are therefore healthy, ‘normal’ individuals' representative of the wild population (Plön et al., 2015). In contrast, samples from stranded animals may not represent the natural population as stranded animals are often sick. Infection by parasites is increasingly being recognised as a cause for strandings and death in dolphins, especially in the case of single strandings (Suárez-González et al., 2024). Unfortunately, very little information is available detailing how infection by parasites is related to odontocete mortality and stranding events in Southern Africa.

The paucity of published data on parasites infecting odontocetes in the Southern African subregion and the need to inform further health studies in small cetaceans from the region prompted the present study. Thus, the aim of this study was two-fold: 1) to identify parasites collected from eight bycaught and stranded odontocete species off Southern Africa (1970–2015); and 2) to investigate trends in infection between stranded and bycaught individuals, sexes, age groups, as well as any temporal changes using prevalence of parasite infection in each of the eight odontocete species and binomial logistic regression models.

## 2 Methods

### 2.1 Study area and sample

**Fig. 1:**
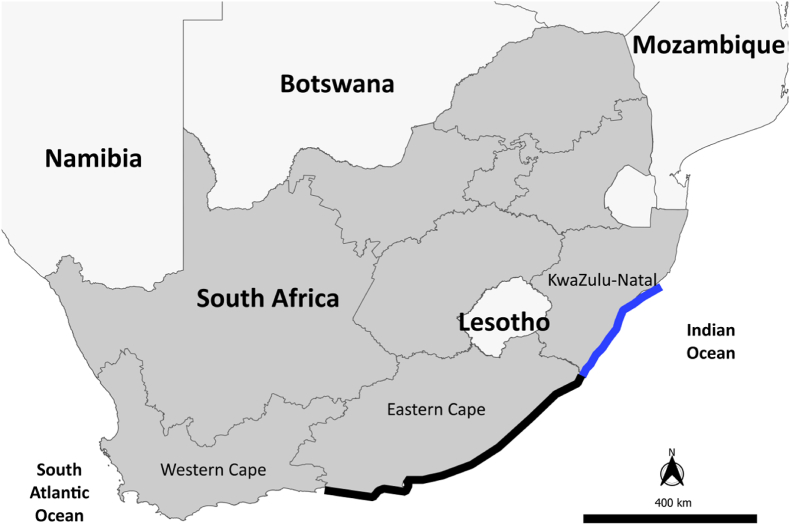
The location of the bycatch from the bather protection nets (BPN), located along the south and central KwaZulu-Natal (KZN) coastline, South Africa, as indicated by the blue line. Strandings were obtained from both the Eastern Cape coastline (represented by the black line) and the KZN coastline combined.

The BPNs, set along the KwaZulu-Natal coastline, South Africa (Fig. 1), are routinely checked every weekday by the KZNSB staff for bycaught animals, including sharks, cetaceans, turtles and seabirds. Live animals are released and recently caught carcasses are removed and transported to the KZNSB laboratory, where detailed biological and morphometrical measurements are recorded. A range of standard tissue samples are also collected for molecular, toxicological and trophic analysis, and pathological examination following a standardized necropsy protocol as of 2010. Prior to 2010, samples were collected opportunistically. The same information and tissue samples, where available, are collected for stranded animals along the Eastern Cape (EC) and KwaZulu-Natal (KZN) coastlines (Fig. 1). These samples are then accessioned and stored at the Graham Ross Marine Mammal Collection located at the Port Elizabeth Museum (PEM; Cockcroft, 1990; Plön et al., 2012).

Parasites were collected from eight bycaught and stranded odontocete species between 1970 and 2015 (Table 1). Systematic collection of parasite samples was only employed since 2010, whereas, prior to 2010, collection of parasite samples was largely opportunistic. To be able to calculate abundance, prevalence etc. for individual host and parasite species, it was therefore assumed that all individual odontocetes with detailed dissection records had been examined for the presence of parasites. Out of a total of 2599 animals examined, 1889 individuals were bycaught, and 710 individuals had stranded. Bycaught odontocete species included *S. plumbea* (n = 171), *T. aduncus* (n = 873), *D. delphis* (n = 823), and *S. coeruleoalba* (n = 22) and stranded odontocete species included *T. aduncus* (n = 248), *D. delphis* (n = 158), *S. coeruleoalba* (n = 90), *S. attenuata* (n = 21), *G. griseus* (n = 105), *K. breviceps* (n = 31), and *K. sima* (n = 57; Table 1). Parasites were stored in glass vials containing either 10 % formalin or 70 % ethanol.

**Table 1:** The number of animals analysed for eight odontocete species from the south-eastern South African coast according to collection method, sex, age class and time interval. B: bycaught; S: stranded; M: male; F: female; N/C: neonate/calf; J/S: juvenile/sub-adult; A: adult; U: unknown.

Dolphin species	Collection method	Sex			Age class				Time intervals				
		M	F	U	N/C	J/S	A	U	1970s	1980s	1990s	2000s	U
*Sousa plumbea*	B (n = 171)	101	66	4	16	68	84	3	2	61	51	56	1
*Tursiops aduncus*	B (n = 873)	414	437	22	295	237	314	27	41	269	276	286	1
	S (n = 248)	113	100	35	99	43	82	24	45	58	67	78	0
*Delphinus delphis*	B (n = 823)	367	442	14	115	123	564	21	18	306	307	192	0
	S (n = 158)	69	64	25	52	13	61	32	22	27	47	62	0
*Stenella coeruleoalba*	B (n = 22)	11	10	1	8	4	9	1	4	6	7	5	0
	S (n = 90)	45	29	16	14	16	48	12	29	23	19	19	0
*Stenella attenuata*	S (n = 21)	11	10	0	3	2	16	0	1	0	8	12	0
*Grampus griseus*	S (n = 105)	43	46	16	30	26	28	21	16	51	31	7	0
*Kogia breviceps*	S (n = 31)	14	13	4	6	5	16	4	15	7	6	3	0
*Kogia sima*	S (n = 57)	23	28	6	6	12	32	7	32	12	3	10	0

Metadata, including, sex, age class, date of death/collection date and collection method (i.e., bycaught vs. stranding) were recorded for each individual (Table 1). Age ranges for each odontocete species were based on total body length (TBL; cm), with individuals categorized into three age classes: neonate/calf, juvenile/sub-adult and adult (Díaz-Delgado et al., 2018; Suárez-González et al., 2024). TBL for age class determination of each species was obtained from the following sources: *S plumbea* (Best, 2007; Plön et al., *submitted*); *T. aduncus* (Cockcroft and Ross, 1990; Best, 2007); *D. delphis* (Ross, 1984; Mendolia, 1989; Young and Cockcroft, 1994); *S. coeruleoalba* (Ross, 1984; Kroese, 1993; Best, 2007; Bishop, 2014); *S. attenuata* (Skinner and Chimimba, 2005; Best, 2007); *G. griseus* (Best, 2007; Hartman, 2018; Plön et al., 2020); *K. breviceps* (Plön, 2004; Best, 2007); and *K. sima* (Plön, 2004; Best, 2007). Time intervals were divided into three 10-year intervals from 1970 to 1999 and one 16-year interval from 2000 to 2015. The last interval encompassed 16 years to ensure enough samples were available for statistical analysis, particularly for stranded dolphin species, such as *G. griseus*, *K. breviceps* and *K. sima*, for which overall sample numbers were low (see Table 1).

### 2.2 Parasite identification

**Fig. 2:**
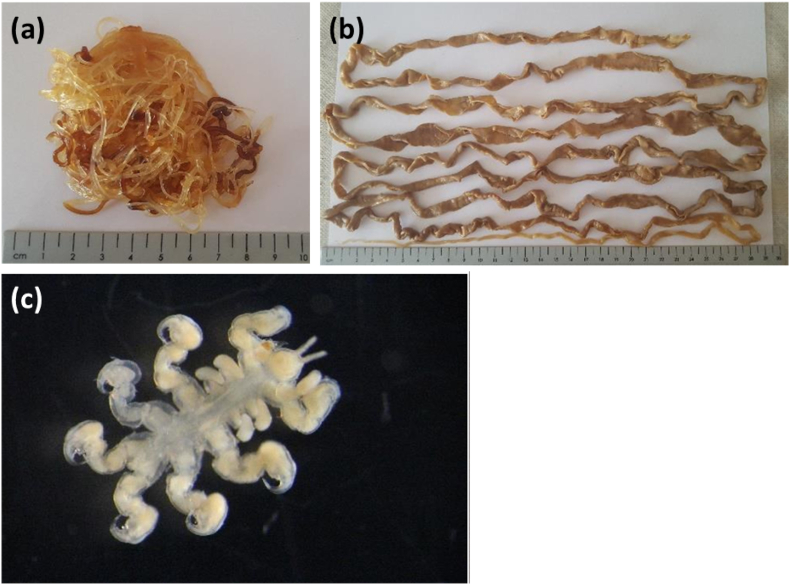
Some parasites identified from odontocetes examined in the present study: (a) *Crassicauda* sp., (b) *Diphyllobothrium* sp. and (c) *Syncyamus* sp.

Since parasite samples were obtained during routine autopsies—often from relatively old carcasses—they were not preserved with taxonomic analysis in mind. Consequently, identification relied solely on morphological characteristics (Fig. 2), following Rohde (2005). Firstly, each individual parasite's taxonomic group was determined (i.e. Cestoda, Nematoda, Trematoda, etc.). Then each specimen's key features, such as mouthparts, digestive organs and reproductive organs, were examined to identify distinguishing characteristics using a LEICA DM500 compound and dissecting microscope. A literature review was conducted to determine whether those parasites have previously been reported for that odontocete species, either in the subregion, or globally.

### 2.3 Statistical analyses

#### 2.3.1 Descriptive statistics: prevalence of parasite infection

Prevalence (%), defined as the number of hosts infected with one or more parasite species divided by the total number of hosts examined, was calculated as described by Bush et al. (1997). Parasite abundance (mean quantity of parasites identified in each host, irrespective of their infection status; calculated as “total number of parasites in all hosts/total number of hosts examined”; Bush et al., 1997) and intensity (mean quantity of parasites identified in hosts that are infected; calculated as “total number of parasites in infected hosts/number of infected hosts”; Bush et al., 1997) could not be calculated as samples were only collected in more detail from 2010.

#### 2.3.2 Modelling approach: presence and absence of parasite infection

Logistic regression analysis was applied to investigate if the probability of parasite presence differs between age class, sex and collection method (bycatch or stranding) within each odontocete species, and if a temporal trend in parasite infections was present during 1970–2015. Data exploration revealed small sample sizes for some species and an imbalanced dataset, with much fewer parasite-presence cases compared to absence cases (Table 2). Such datasets are problematic for traditional logistic regression methods, such as Generalized Linear Models (GLMs), often leading to models with low statistical power and bias in the results (Bissonette, 1999; Wisz et al., 2008; Salas-Eljatib et al., 2018). To overcome this challenge, the Firth's bias-reduced logistic regression is used as the modelling approach in this study, as this method has been found to sufficiently address potential bias and uncertainties associated with small sample sizes and imbalanced datasets (Firth, 1993; Heinze and Schemper, 2002; Salas-Eljatib et al., 2018).

**Table 2:** Sample size per species (by collection method) available for the regression models, after excluding individuals with unknown age class, sex or date of collection from analysis. “Presence” indicates number of individuals infected with parasites, regardless of the number or species of parasites found. “Absence” indicates number of individuals with no parasites.

Species	Before			After		
	Total	Absence	Presence	Total (% excluded)	Absence (% excluded)	Presence (% excluded)
*D. delphis* (bycatch)	823	797	26	796 (3 %)	771 (3 %)	25 (4 %)
*D. delphis* (stranding)	158	147	11	116 (27 %)	105 (28 %)	11 (0 %)
*T. aduncus* (bycatch)	873	802	71	836 (4 %)	765 (5 %)	71 (0 %)
*T. aduncus* (stranding)	248	228	20	208 (16 %)	188 (18 %)	20 (0 %)
*S. coeruleoalba* (bycatch)	22	19	3	21 (5 %)	18 (5 %)	3 (0 %)
*S. coeruleoalba* (stranding)	90	74	16	70 (20 %)	55 (26 %)	15 (6 %)
*S. plumbea* (bycatch)	171	152	19	165 (4 %)	147 (3 %)	18 (5 %)
*G. griseus* (stranding)	105	96	9	76 (28 %)	68 (29 %)	8 (11 %)
*K. breviceps* (stranding)	31	26	5	26 (16 %)	21 (19 %)	5 (0 %)
*K. sima* (stranding)	57	48	9	49 (14 %)	40 (17 %)	9 (0 %)
*S. attenuata* (stranding)	21	18	3	21 (0 %)	18 (0 %)	3 (0 %)

A Firth's logistic regression model was fitted using a binomial distribution family with the *logistf* package in R (version 4.2.1; Heinze et al., 2023). One model was built for each species. Additionally, for species with both bycatch and stranding data, three models were built: a full model (bycatch and stranding data combined), a bycatch-only model and a stranding-only model. Within each odontocete species, the presence (1) and absence (0) of parasites in each individual is used as the response variable, regardless of the number or species of parasites found. Age class, sex, time interval and collection method (if applicable) were included as categorical explanatory variables. No interaction terms were included in the model, as the primary objective was to assess main effects. Individuals with unknown age class, sex or date of collection (n = 215; 8 %) were excluded from analysis (Table 2).

## 3 Results

### 3.1 Parasite identification

Seventeen parasite taxa were identified in the eight odontocete species examined (Table 3). Two parasite taxa could only be identified to family level: Aegidae and Cymothoidae. Aegidae was found to be present in *T. aduncus* and Cymothoidae infected only *S. plumbea*.

**Table 3:** List of parasite species identified from eight bycaught and stranded odontocetes (1970–2015) from south-eastern South Africa.

Parasite taxa	Tissue/Organ infected	Host
Aegidae	External	*T. aduncus, D. delphis*
*Anisakis* sp.	Stomach	*S. plumbea, T. aduncus, D. delphis, S. coeruleoalba,* *K. breviceps, K. sima*
*Conchoderma auritum*	External	*T. aduncus*
*Coronula diadema*	External	*S. plumbea*
*Crassicauda* sp.	Muscle, sinuses	*T. aduncus, G. griseus, K. breviceps*
Cymothoidae	External	*S. plumbea*
*Diphyllobothrium* sp.	Intestine	*T. aduncus, D. delphis, S. coeruleoalba*
*Halocercus* sp.	Lungs	*S. plumbea, T. aduncus, D. delphis, S. coeruleoalba, K. sima*
*Monorgyma grimaldii*	Ovary, testis	*T. aduncus, D. delphis, S. coeruleoalba, S. attenuata*
*Nasitrema* sp.	Sinuses	*S. plumbea, T. aduncus, D. delphis*
*Notomegabalanus algicola*	External	*T. aduncus*
*Pennella balaenoptera*	External	*K. breviceps, K. sima*
*Phyllobothrium delphini*	Blubber	*S. plumbea, T. aduncus, D. delphis, S. coeruleoalba, S. attenuata, K. breviceps, K. sima*
*Pseudoterranova* sp.	Stomach	*T. aduncus, D. delphis*
*Syncyamus* sp.	Blowhole	*T. aduncus, D. delphis, S. coeruleoalba*
Unidentified cestode	Intestine	*D. delphis, S. coeruleoalba*
*Xenobalanus globicipitis*	Skin	*S. plumbea, T. aduncus, S. coeruleoalba, G. griseus*

Seven parasites were identified to genus level, namely *Anisakis* sp., *Crassicauda* sp., *Diphyllobothrium* sp., *Halocercus* sp., *Nasitrema* sp., *Pseudoterranova* sp., and *Syncyamus* sp., affecting all odontocete species, except *S. attenuata* (Table 3). *Anisakis* sp., a parasite found in the stomach, infected almost all odontocete species (six species), followed by *Halocercus* sp., which was found in the lungs of five host species. *Crassicauda* sp., *Diphyllobothrium* sp., *Nasitrema* sp., and *Syncyamus* sp. were each present in three odontocete species, occupying various tissues/organs, such as muscle, sinuses, intestine, stomach and blowhole (Table 3). *Pseudoterranova* sp. was only found in the stomachs of two odontocete species: *T. aduncus* and *D. delphis*.

Eight parasites were identified to species level (Table 3). External parasites, *Conchoderma auritum* and *Notomegabalanus algicola* were only found in *T. aduncus, while Coronula diadema* only infected *S. plumbea*. *Phyllobothrium delphini* was found in the blubber of seven odontocete species, with the exception of *G. griseus*. *Monorgyma grimaldii* infected the ovaries/testes of four odontocete species, namely, *T. aduncus, D. delphis, S. coeruleoalba* and *S. attenuata* and *Xenobalanus globicipitis* was found on the skin of *S. plumbea, T. aduncus, S. coeruleoalba* and *G. griseus* (Table 3).

One unidentified cestode infected the intestines of *D. delphis* and *S. coeruleoalba,* while *Pennella balaenoptera*, an external parasite, was present only in the two *Kogia* species (Table 3).

### 3.2 Descriptive statistics: prevalence (%)

#### 3.2.1 Parasite prevalence per host species

For parasite identification, 192 out of the 2599 bycaught and stranded individuals contained one or more parasite groups (Table 4). With the exception of *G. griseus* (8.57 %), the offshore odontocete species (*S. coeruleoalba*, *S. attenuata*, *K. breviceps* and *K. sima*) appeared to have higher parasitic prevalences (>13 %) than the more coastal species, such as *S. plumbea*, *T. aduncus*, and *D. delphis* (<12 %; Table 4).

**Table 4:** Prevalence (%) of parasitic infection in eight bycaught and stranded odontocete species from the south-eastern coastline of South Africa (1970–2015).

	Collection method	Parasitized animals	Total examined	Prevalence (%)
*S. plumbea*	Bycaught	19	171	11.11
*T. aduncus*	Bycaught	71	873	8.13
	Stranded	20	248	8.06
*D. delphis*	Bycaught	26	823	3.16
	Stranded	11	158	6.96
*S. coeruleoalba*	Bycaught	3	22	13.64
	Stranded	16	90	17.78
*S. attenuata*	Stranded	3	21	14.29
*G. griseus*	Stranded	9	105	8.57
*K. breviceps*	Stranded	5	31	16.13
*K. sima*	Stranded	9	57	15.79

Among the four bycaught species, *S. coeruleoalba* had the highest prevalence of infection (13.64 %), followed by *S. plumbea* (11.11 %), *T. aduncus* (8.13 %) and *D. delphis* (3.16 %), which had the lowest parasitic prevalence (Table 4). Similarly, of the seven stranded species, *S. coeruleoalba* had the highest parasitic prevalence (17.78 %), and *D. delphis* exhibited the lowest prevalence (6.96 %). The other stranded species, in order of decreasing parasitic prevalence, were *K. breviceps* (16.13 %), *K. sima* (15.79 %), *S. attenuata* (14.29 %), *G. griseus* (8.57 %), and *T. aduncus* (8.06 %; Table 4).

#### 3.2.2 Parasite species-specific prevalence in both bycaught and stranded animals

**Fig. 3:**
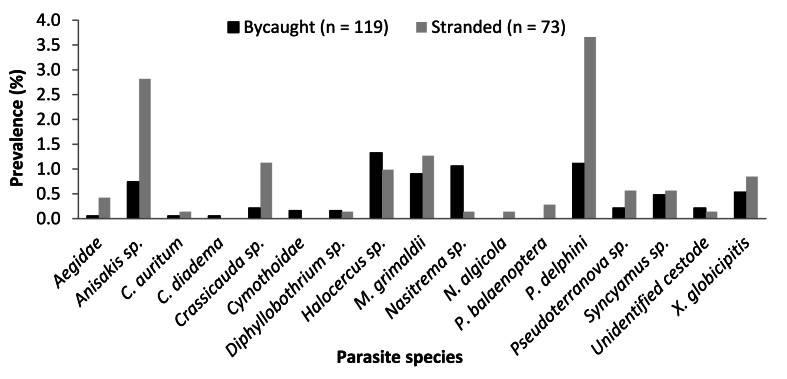
Overall prevalence (%) of parasitic infection between bycaught (n = 119) and stranded (n = 73) odontocete species from the south-eastern coastline of South Africa (1970–2015).

The overall parasitic prevalence (%) was generally low (<4 %) for both bycaught and stranded odontocetes between 1970 and 2015 (Fig. 3). *T. aduncus*, *D. delphis* and *S. coeruleoalba* were the only species which had both bycaught and stranded individuals (see section 3.2.3). Some parasite species were only present in either bycaught or stranded animals: Cymothoidae and *C. diadema* were only found in bycaught animals, while *N. algicola* and *P. balaenoptera* were only identified in stranded animals (Fig. 3). Amongst bycaught animals, *Halocercus* sp. had the highest prevalence (1.32 %), followed by *P. delphini* (1.11 %) and *Nasitrema* sp. (1.06 %). These were the only parasites in the bycaught sample, which had a prevalence higher than 1 % (Fig. 3). Aegidae, *C. auritum* and *C. diadema* had the lowest prevalence in the bycaught sample, each with a prevalence of 0.05 % (Fig. 3). In contrast, in stranded animals, *P. delphini* had the highest prevalence (3.66 %), followed by *Anisakis* sp. (2.82 %), *M. grimaldii* (1.27 %) and *Crassicauda* sp. (1.13 %), all with prevalences above 1 %. *C. auritum, N. algicola,* Diphyllobothrium sp., and *Nasitrema* sp. had the lowest prevalence in the stranded sample, each with a prevalence of 0.14 % (Fig. 3).

Overall, out of the 13 parasite species identified in both bycaught and stranded animals, prevalence was generally higher in stranded animals, except for four parasite species (*Diphyllobothrium* sp., *Halocercus* sp., *Nasitrema* sp., and the unidentified cestode), which were more prevalent in bycaught individuals (Fig. 3).

**Fig. 4:**
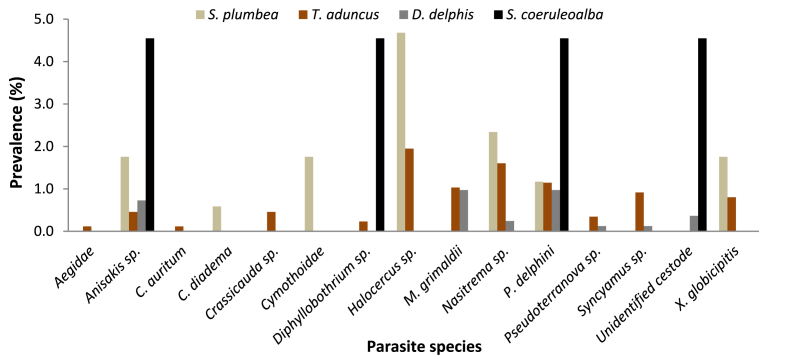
Prevalence (%) of parasitic infection in bycaught odontocete species from the south-eastern coastline of South Africa (1970–2015).

Bycaught animals were infected by 15 parasite taxa in total (Fig. 4). *S. coeruleoalba* and *S. plumbea* individuals appeared to have the highest prevalence of parasitic infections out of the bycaught animals, followed by *T. aduncus* and *D. delphis* (Fig. 4). Among the bycaught animals, *T*. *aduncus* individuals contained the highest number of parasite species (n = 12), followed by *S. plumbea* (n = 9), *D. delphis* (n = 7) and *S. coeruleoalba* (n = 4). Aegidae (0.11 %), *C. auritum* (0.11 %) and *Crassicauda* sp. (0.46 %) were only found in bycaught *T. aduncus* individuals, while *C. diadema* (0.58 %) and Cymothoidae (1.75 %) were only found in bycaught *S. plumbea* individuals (Fig. 4). In contrast, *Anisakis* sp. and *P. delphini* were found infecting all four bycaught dolphin species, with *S. coeruleoalba* (4.55 % for both parasite species) and *S. plumbea* (1.75 % and 1.17 %, respectively) having the highest prevalence of both parasite species (Fig. 4).

**Fig. 5:**
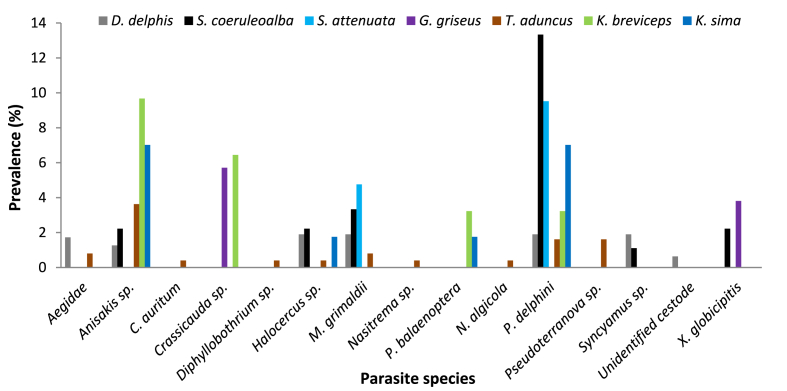
Prevalence (%) of parasitic infection in stranded odontocete species from the south-eastern South African coastline (1970–2015).

Stranded animals were infected by 15 parasite taxa in total (Fig. 5). Among the stranded animals, *T*. *aduncus* individuals once again contained the highest number of parasite species (n = 10), followed by *D. delphis* (n = 8) and *S. coeruleoalba* (n = 6). Each *Kogia* sp. was infected with four parasite taxa, while *S. attenuata* and *G. griseus* individuals were only infected by two parasite taxa (Fig. 5).

*C. auritum* (0.40 %), *Diphyllobothrium* sp. (0.40 %), *Nasitrema* sp. (0.40 %), *N. algicola* (0.40 %), and *Pseudoterranova* sp. (1.61 %) were only found in stranded *T. aduncus* individuals, while the unidentified cestode (0.63 %) was only found in stranded *D. delphis* individuals. *P. delphini* was found in stranded individuals of all odontocete species, with the exception of *G. griseus*. *S. coeruleoalba* the highest prevalence of *P. delphini* (13.33 %), one of the highest parasite prevalences recorded among both the bycaught and stranded animals (Fig. 5).

#### 3.2.3 Parasite species-specific prevalence in bycaught vs. stranded animals

**Fig. 6:**
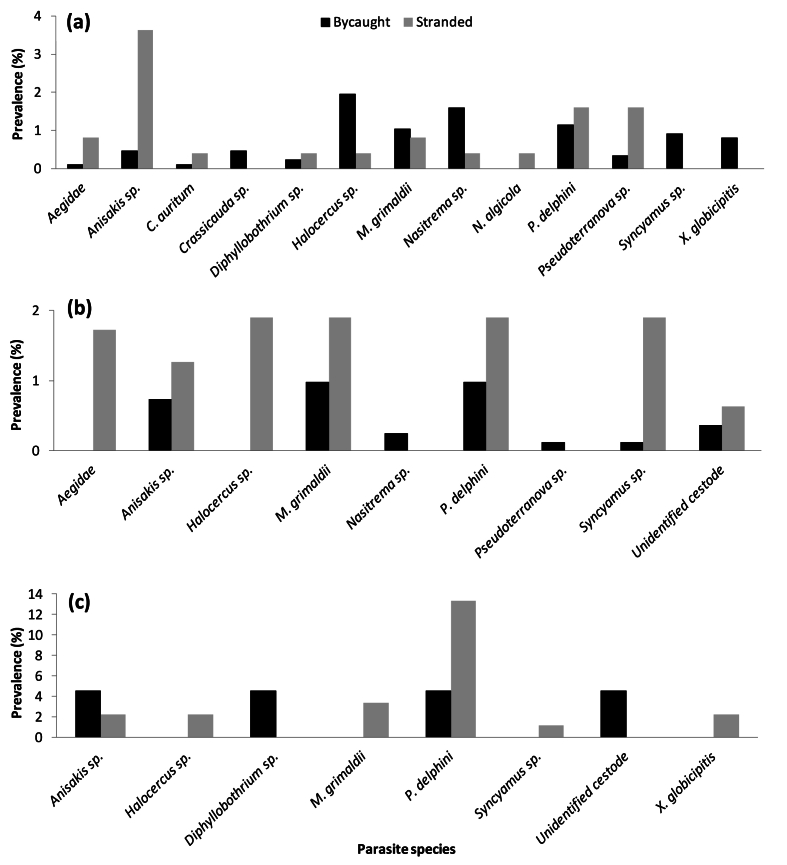
Prevalence (%) of parasitic infection in three bycaught and stranded odontocete species from the south-eastern South African coastline (1970–2015); where (a) *T. aduncus*; (b) *D. delphis* and (c) *S. coeruleoalba*.

For those species, for which both bycaught and stranded individuals were investigated, namely *T. aduncus* (Fig. 6a), *D. delphis* (Fig. 6b) and *S. coeruleoalba* (Fig. 6c), parasitic prevalence (%) was compared between bycaught and stranded animals.

Bycaught and stranded *T. aduncus* individuals were infected with the highest overall number of parasite species (13 different species) out of the eight odontocete species examined (Fig. 6a). *C. auritum* and *N. algicola* were only found in this host species, with *N. algicola* only infecting stranded *T. aduncus* individuals. In contrast, *Crassicauda* sp., *Syncamus* sp. and *X. globicipitis* only affected bycaught individuals and were not found in stranded *T. aduncus* (Fig. 6a). Among the bycaught *T. aduncus* individuals, *Halocercus* sp. had the highest prevalence (1.95 %), while Aegidae and *C. auritum* had the lowest prevalence (0.11 % each; Fig. 6a). In the stranded *T. aduncus* sample, *Anisakis* sp. had the highest prevalence (3.63 %), while the lowest prevalence was shared between five parasite species, namely *C. auritum*, *Diphyllobothrium* sp., *Halocercus* sp., *Nasitrema* sp. and *N. algicola*, each with a prevalence of 0.40 % (Fig. 6a).

Bycaught and stranded *D. delphis* had nine parasite species (Fig. 6b), with *Nasitrema* sp. and *Pseudoterranova* sp. only being found in the bycaught sample and Aegidae and *Halocercus* sp. infecting only stranded individuals. In bycaught *D. delphis*, *M. grimaldii* and *P. delphini* had the highest prevalence of 0.97 % each, while *Pseudoterranova* sp. and *Syncamus* sp. had the lowest prevalence of 0.12 % each (Fig. 6b). The stranded *D. delphis* individuals had a generally higher prevalence of parasitic infection when compared to the bycaught individuals, with four parasite species (*Halocercus* sp., *M. grimaldii*, *P. delphini* and *Syncamus* sp.) having the highest prevalence value of 1.90 % (Fig. 6b). The lowest prevalence of 0.63 % found in the stranded *D. delphis* sample belonged to the unidentified cestode, the only parasite species with a prevalence lower than 1 % amongst the stranded individuals (Fig. 6b).

Eight parasite species were found in the bycaught and stranded *S. coeruleoalba* sample (Fig. 6c). Among the bycaught *S. coeruleoalba* individuals, only four parasite species (*Anisakis* sp., *Diphyllobothrium* sp., *P. delphini* and the unidentified cestode) were found, each with a prevalence of 4.55 % (Fig. 6c). *Diphyllobothrium* sp. and the unidentified cestode were not found in the stranded *S. coeruleoalba* individuals. *P. delphini* had the highest prevalence (13.33 %) in the stranded *S. coeruleoalba* sample, while *Syncyamus* sp. had the lowest prevalence (1.11 %; Fig. 6c).

#### 3.2.4 Prevalence by sex, age class and time intervals

Parasitic prevalence (%) according to sex, age class and time intervals for the eight bycaught and stranded odontocete species showed variation both between categories and host species (Table 5). For sex, the males of most host species had a higher prevalence (range: 3.27 %–21.43 %) when compared to females (range: 2.94 %–15.38 %), with the exception of bycaught *S. plumbea* (12.12 % for females vs. 10.89 % for males), stranded *D. delphis* (9.38 % for females vs. 7.25 % for males) and both bycaught and stranded *S*. *coeruleoalba* (20.00 % and 27.59 % for females, respectively, and 9.09 % and 17.39 % for males), where females had a higher prevalence than males (Table 5).

**Table 5:** Prevalence (%) of parasitic infection, according to sex, age class and time intervals, in eight bycaught and stranded odontocete species from south-east South Africa (1970–2015). The highest prevalence of each category is highlighted in bold. *S.p*: *Sousa plumbea*; *T.a*: *Tursiops aduncus*; *D.d*: *Delphinus delphis*; *S.c*: *Stenella coeruleoalba*; *S.a*: *Stenella attenuata*; *G.g*: *Grampus griseus*; *K.b*: *Kogia breviceps*; *K.s*: *Kogia sima*.

Categories			*S.p*	*T.a*	*D.d*	*S.c*	*S.a*	*G.g*	*K.b*	*K.s*
Sex	**Bycaught**	**Male**	10.89	**9.18**	**3.27**	9.09	–	–	–	–
		**Female**	**12.12**	7.55	2.94	**20.00**	–	–	–	–
	**Stranded**	**Male**	–	**10.62**	7.25	17.39	**18.18**	**11.63**	**21.43**	
		**Female**	–	8.00	**9.38**	**27.59**	10.00	8.70	15.38	
Age class	**Bycaught**	**Neonate/calf**	**18.75**	8.81	1.74	**25.00**	–	–	–	–
		**Juvenile/sub-adult**	11.76	**11.21**	**4.07**	0	–	–	–	–
		**Adult**	9.52	6.10	3.37	7.69	–	–	–	–
	**Stranded**	**Neonate/calf**	–	4.04	**9.62**	0	0	3.33	0	16.67
		**Juvenile/sub-adult**	–	9.30	7.69	18.75	0	**19.23**	**40.00**	**25.00**
		**Adult**	–	**14.63**	8.20	**25.00**	**18.75**	7.14	18.75	15.63
Time intervals	**Bycaught**	**1970–1979**	0	0	0	0	–	–	–	–
		**1980–1989**	8.20	11.99	**4.90**	16.67	–	–	–	–
		**1990–1999**	0	1.45	0.65	0	–	–	–	–
		**2000–2015**	**23.21**	**12.24**	4.69	**40.00**	–	–	–	–
	**Stranded**	**1970–1979**	–	2.22	4.55	3.45	0	0	0	3.13
		**1980–1989**	–	8.62	**18.52**	**21.74**	**6.00**	**11.76**	**42.86**	25.00
		**1990–1999**	–	4.48	0	21.05	3.00	9.68	33.33	33.33
		**2000–2015**	–	**14.10**	8.06	31.58	0	0	0	**40.00**

Examining prevalence in relation to age class of the host, it was highest in *S. plumbea* and *S*. *coeruleoalba* neonates/calves (18.75 % and 25.00 %, respectively) and in *T. aduncus* and *D. delphis* juveniles/sub-adults (11.21 % and 4.07 %, respectively; Table 5). Among the bycaught animals, adults had the lowest prevalence out of the three age classes. No parasites were recorded in the bycaught *S. coeruleoalba* juvenile/sub-adult age class. Among the stranded animals, prevalence varied between the age classes of the eight odontocete species (Table 5). Prevalence was highest in *D. delphis* neonates/calves (9.62 %); *G*. *griseus* (19.23 %), *K*. *breviceps* (40.00 %) and *K*. *sima* (25.00 %) juveniles/sub-adults and *T. aduncus* (14.63 %) and *S. coeruleoalba* (25.00 %) adults (Table 5). No parasites were recorded in the neonate/calf age class of stranded *S. coeruleoalba*, *S. attenuata* and *K. breviceps*; as well as in the juvenile/sub-adult group of *S. attenuata* (Table 5).

In the bycaught sample, the highest prevalence was recorded in the 2000–2015 interval for *S. plumbea* (23.21 %), *T. aduncus* (12.24 %) and *S. coeruleoalba* (40.00 %); whereas *D. delphis* had the highest parasitic prevalence in the 1980 to 1989 time interval (4.90 %; Table 5). The lowest prevalence was recorded in the 1990–1999 interval. No parasites were recorded in the 1970s for all bycaught species and in the 1990 to 1999 interval for bycaught *S. plumbea* and *S. coeruleoalba* (Table 5). For the stranded individuals, the highest parasitic prevalence appeared in the 1980–1989 and 2000–2015 intervals for all species. Prevalence for *D. delphis* (18.52 %), *S. attenuata* (6.00 %)*, G*. *griseus* (11.76 %) and *K. breviceps* (42.86 %) was highest in 1980–1989 and highest for *T. aduncus* (14.10 %), *S. coeruleoalba* (31.58 %) and *K*. *sima* (40.00 %) in 2000–2015 (Table 5). The lowest prevalence was recorded in the 1970–1979 interval. No parasites were recorded for stranded *S. attenuata, G*. *griseus* and *K. breviceps* in both the 1970 to 1979 and 2000 to 2015 time intervals. In addition, no parasites were recorded for stranded *D. delphis* in 1990–1999 (Table 5).

### 3.3 Presence and absence of parasites: trends in sex, age class, time intervals and collection method

The presence and absence of parasite species in each odontocete host was evaluated using the Firth's bias-reduced logistic regression to determine whether any trends in parasitic infection existed between the different sexes, age classes, time intervals and collection methods (Table 6).

**Table 6:** Summary of the main results from the Firth's logistic regression models. For odontocete species with both bycaught and stranded animals, three models were built: (1) full model (combined bycaught and stranded animals), (2) bycatch-only model, and (3) stranding-only model. Models were based on the presence and absence of parasite species in each species. Only models and predictors for which a significant result (*p* > 0.05) was obtained are shown (see Supplementary Material 1 for complete results).

Species (model)	Predictor		
	Age class	Time interval	Collection method
*Delphinus dephis* (full model)	–	–	✓ Stranded individuals have higher parasite presence
*Tursiops aduncus* (full model)	–	✓ Parasite presence higher in 1980–1989 and 2000–2015	–
*Tursiops aduncus* (bycatch)	–	✓ Parasite presence higher in 1980–1989 and 2000–2015	–
*Tursiops aduncus* (stranding)	✓ Parasite presence lower in neonates/calves	–	–
*Stenella coeruleoalba* (full model)	–	✓ Parasite presence higher in 2000–2015	–
*Stenella coeruleoalba* (stranding)	✓ Parasite presence lower in neonates/calves	✓ Parasite presence higher in 1990–1999 and 2000–2015	–
*Kogia breviceps* (stranding)	–	✓ Parasite presence higher in 1980–1989 and 1990–1999	–
*Kogia sima* (stranding)	–	✓ Parasite presence higher in 1980–1989, 1990–1999 and 2000–2015	–

Out of the three odontocete species (*D. delphis*, *T. aduncus* and *S. coeruleoalba*) with both bycaught and stranded animals, the collection method was only significant for *D. delphis* (full model), where stranded animals have a significantly higher probability of parasite presence than bycaught animals (*p* = 0.003).

In terms of differences between age classes, the probability of parasite presence was significantly lower for the neonates/calves of stranded *T. aduncus* (*p* = 0.01) and stranded *S. coeruleoalba* (*p* = 0.02), only when tested using the stranding-only models for both species. In all other species and collection methods, there was no significant differences in the probability of parasite presence between the age classes.

No differences were found in the probability of parasite presence between the sexes of all species.

A temporal trend was detected in the probability of parasite presence for *T. aduncus*, *S. coeruleoalba*, *K. breviceps* and *K. sima*. Both the full model and bycatch-only model for *T. aduncus* showed that the probability of parasite presence was significantly higher during 1980–1989 (full model: *p* = 0.002; bycatch-only model: *p* = 0.01) and 2000–2015 (full model: *p=*0.001; bycatch-only model: *p* = 0.01). This temporal trend was not detected in the stranding-only model for *T. aduncus*, suggesting that the trend was only true for bycaught animals and that the full model was unable to detect the difference in temporal pattern between the bycatch and stranding datasets. Similarly, a temporal pattern for *S. coeruleoalba* was detected only in the full model and stranding-only model, but not in the bycaught model. In this case, there was also a difference in the results of these two models. In the full model, the probability of parasite presence in *S. coeruleoalba* was significantly higher only during 2000–2015 (*p* = 0.01). On the other hand, the probability of parasite presence in stranded *S. coeruleoalba* was significantly higher during 1990–1999 (*p* = 0.04) and 2000–2015 (*p* = 0.04). This suggests that the full model for *S. coeruleoalba* was only able to partially detect the temporal pattern within the dataset but was also unable to differentiate between the underlying patterns of the stranding and bycatch datasets. In both cases, the dataset of one collection method has a lower sample size compared to the other: there were fewer bycaught *S. coeruleoalba* (n = 21) than stranded animals (n = 70), and fewer stranded *T. aduncus* (n = 208) than bycaught animals (n = 836; Table 2). This large differences in sample size may have contributed to the inability of the full models of both species to detect the different temporal pattern between collection methods. For *K. breviceps*, where only stranded animals were available for analysis, the probability of parasite presence was significantly higher in 1980–1989 (*p* = 0.01) and 1990–1999 (*p* = 0.02). It should be noted that the very small sample size (n = 26) for *K. breviceps* may have reduced the explanatory power of the model. The probability of parasite presence in stranded *K. sima* was significantly higher in 1980–1989 (*p* = 0.015), 1990–1999 (*p* = 0.04) and 2000–2015 (*p* = 0.004). However, there was a large difference in both the number of strandings and infected animals in 1970–1979 alone (one infected animals out of 27 strandings) compared to the later years (1980–1989: three infected animals out of 10 strandings; 1990–1999: one infected animal out of three strandings; 2000–2015: four infected animals out of nine strandings). This begs the question of whether the temporal pattern detected by the model is a true biological trend or simply an artefact of small sample size and/or imbalanced data.

None of the predictors tested were significant for *S. plumbea*, *S. attenuata* and *G. griseus*.

## 4 Discussion

### 4.1 Parasites infecting odontocetes off southern Africa

Globally, numerous studies have been conducted to investigate the interactions between parasite species and their marine mammal hosts (e.g., Mignucci-Giannoni et al., 1998; Oliveira et al., 2011; Suárez-González et al., 2024). In South Africa, research on the parasitic fauna in marine mammals has been limited, with most studies focusing on parasites found in either bycaught animals or stranded carcasses, which are often in an advanced stage of decomposition (e.g., Ross et al., 1994; Best, 2007; Lane et al., 2014). This study used a long-term dataset (1970–2015) of eight odontocete species bycaught and stranded along the south-eastern coastline of South Africa to determine the prevalence of parasites. It provided a first record of parasites infecting small cetaceans for the subregion, highlighting the value of museum collections for biological studies. Out of the 2599 individual odontocetes, only 192 specimens had parasites present. Numerous parasites were recorded for the first time in several of the odontocete species examined from South Africa.

Seven genera of parasites were found to infect bycaught *S. plumbea* (Table 3; Fig. 4), with only three parasite species being formally reported in previous studies (Ross et al., 1994; Lane et al., 2014; Van Bressem et al., 2020). An amphipod, *Syncyamus aequus*, is the only external parasite recorded, which was obtained from two humpback dolphins off the KwaZulu-Natal coastline (Ross et al., 1994) and, the presence of *Halocercus* sp. has also been identified in the bronchi of this species in southern Africa (Lane et al., 2014). More recently, Van Bressem et al. (2020) examined the skulls of bycaught and stranded humpback dolphins in South Africa and found that 13 % of bycaught animals were affected by cranial crassicaudiasis, a condition caused by the invasion of *Crassicauda* spp. (Nematoda) into the cranial sinuses of host species. *Halocercus* sp. was the only parasite species, which was previously reported for the subregion, that was also found in this study. *C. diadema*, *P. delphini*, *X. globicipitis*, *Anisakis* sp., *Nasitrema* sp. and Cymothoidae were all new parasite records for *S. plumbea* for the subregion.

Thirteen parasite species were found in bycaught and stranded *T. aduncus* individuals, the host species with the highest number of parasites out of the eight odontocetes analysed (Table 3; Fig. 4, Fig. 5, Fig. 6a). A number of parasites have previously been identified for this host species in the sub-region. These include the lungworm *Halocercus* sp. (Lane et al., 2014), the barnacle *Xenobalanus* sp. (Ross, 1984; Best, 2007; Lane et al., 2014), nematodes from the Anisakidae family and *Crassicauda* sp. (Lane et al., 2014) and three unidentified cyamids recorded on the edge of the blowhole (Ross, 1984). Four parasite species previously reported for *T. aduncus* were also found in this study (*Halocercus* sp., *X. globicipitis, Anisakis* sp. and *Crassicauda* sp.), while *C. auritum*, *M. grimaldii*, *N. algicola*, *P. delphini*, *Diphyllobothrium* sp., *Nasitrema* sp., *Pseudoterranova* sp., *Syncyamus* sp., and Aegidae being new species reported in this study for the sub-region.

Bycaught and stranded *D. delphis* contained nine parasite species (Table 3; Fig. 4, Fig. 5, Fig. 6b). *Xenobalanus* sp., found on the flippers or flukes, and *Syncyamus aequus*, found around the blowhole, eyes, throat, upper lip, lower jaw and side of the head, are the only external parasites reported for this species in the sub-region (Best, 2007). *Syncyamus* sp. was the only previously reported parasite species in the subregion found in this study, indicating that *M. grimaldii*, *P. delphini*, *Anisakis* sp., *Halocercus* sp., *Nasitrema* sp., *Pseudoterranova* sp., Aegidae and the unidentified cestode are all newly reported parasite species infecting *D. delphis* in the subregion.

Eight parasite species were found in the bycaught and stranded *S. coeruleoalba* individuals in this study (Table 3; Fig. 4, Fig. 5, Fig. 6c); only *Xenobalanus* sp., present on the flippers, and *Syncyamus* sp., found in the blowhole of several individuals, had previously been recorded in the southern African region (Ross, 1984). Out of the eight parasite species found in this study, six parasite species, namely, *M. grimaldii*, *P. delphini*, *Anisakis* sp., *Diphyllobothrium* sp., *Halocercus* sp. and the unidentified cestode, are newly reported parasite species for the subregion.

In this study, only two parasite species (*P. delphini* and *M. grimaldii*) were found in *S. attenuata* (Table 3; Fig. 5). *Xenobalanus* sp., *Syncyamus* sp., and *C. auritum* have previously been recorded on the flukes, flippers and teeth of several individuals in the sub-region (Ross, 1984; Perrin, 1969). *Crassicauda* sp. (Perrin, 1969) and *Anisakis* sp. (Cavallero et al., 2001) have also both been recorded in this species for the sub-region. Thus, both parasite species found in this study, *P. delphini* and *M. grimaldii*, are newly reported parasite species found in *S. attenuata* in the subregion.

Only two parasitic genera (*X. globicipitis* and *Crassicauda* sp.) were found in *G. griseus* (Table 3; Fig. 5), which had both been previously reported for the species (Dailey and Stroud, 1978; Best, 2007). Previous studies have found *Xenobalanus* sp. and *Scutocyamus* sp. on the dorsal fins, flippers and tails of this species in the sub-region (Best, 2007). *Penella* sp., *Monorgyma* sp., *Crassicauda* sp. and *Anisakis* sp. have also been reported for this host species in southern Africa (Dailey and Stroud, 1978).

Four parasite species were found to infect both *K. breviceps* (infected with *P. balaenoptera*, *P. delphini*, *Anisakis* sp. and *Crassicauda* sp.) and *K. sima* (infected with *P. balaenoptera*, *P. delphini*, *Anisakis* sp. and *Halocercus* sp.) in this study (Table 3; Fig. 5). To the best of our knowledge, there are no previous records of parasitic infection in *K. breviceps* and *K. sima* from the southern African region, making this the first record in the sub-region.

### 4.2 Parasite specific infection: infection sites and health concerns

The external parasites found in this study, namely, *C. auritum*, *C. diadema*, *N. algicola*, *P. balaenoptera*, *X. globicipitis*, and those belonging to the Aegidae and Cymothoidae families, are all commonly found attached to the skin, blowhole and even the teeth of their host. These external parasite species had some of the lowest prevalences amongst host species (Fig. 3, Fig. 4, Fig. 5, Fig. 6), which is probably not a true representation of their numbers as external parasites can often become dislodged during collection of stranded host species or during their struggle in the nets in the case of bycaught hosts (Nagasawa, 1985; Williams et al., 1991). Most of these external parasites were only found to infect one or two host species (Table 3), with the exception of *X. globicipitis*, which appears to infect more host species (Table 3) and have a higher prevalence than the other external parasites (Fig. 3). This may be due to the fact that *X. globicipitis* is specialised in living on cetaceans, most commonly found around the edges of flukes and fins (Kane et al., 2008).

*Halocercus* sp., a lungworm, had the highest prevalence amongst the bycaught individuals (Fig. 3), more specifically, in bycaught *S. plumbea* (Fig. 4), which is of concern as these parasites can cause severe health problems. These include osseous lesions in the cranial sinuses, blocked airways, verminous pneumonia and secondary bacterial infections, all of which can lead to the stranding or even death of their hosts (Measures, 2001). With *S. plumbea* being the only endangered resident marine mammal in South African waters (Braulik et al., 2015, 2023; Plön et al., 2016), this factor will need to be taken into consideration for future conservation and management plans. *Anisakis* sp. is a very common nematode infecting the stomachs of marine mammals (Suárez-González et al., 2024). In this study, *Anisakis* sp. infected six out of the eight odontocete species investigated (Table 3). This nematode can not only cause inflammation, ulcers, hemorrhages and necrotic lesions in the stomachs of cetaceans (Suárez-González et al., 2024), but it is of zoonotic importance as it poses a risk to human health through ingestion of larvae in raw or undercooked fish (Mattiucci and Nascetti, 2006; Shamsi, 2019). *P. delphini* had the highest prevalence among the stranded host species (Fig. 3) but also infected seven out of the eight host species investigated in this study (Table 3). *P. delphini* forms cysts in the sub-cutaneous blubber of cetaceans, which can cause a localised lymphoplasmacytic response in the host species and affect the host's ability to swim (Dailey and Walker, 1978; Norman, 1997).

The unidentified cestode species in our study, found in the intestines of two dolphin species, *D. delphis* and *S. coeruleoalba* (Table 3), was suggested to be *Hymenolepsis nana*, a tapeworm which is typically found in the intestines of humans and rodents (Ito and Budke, 2021). Although it may be the first time this parasite is reported to occur in cetaceans, it is conceivable that oocysts deposited in the faeces of the host species on land may wash off into the ocean and affect cetaceans this way, as is considered the likely mode of transmission for *Toxoplasma gondii*. *T. gondii* oocysts are typically found in cats and have the potential to enter the marine environment via runoff and sewage discharge. These oocysts may subsequently be consumed by marine invertebrates, which are then preyed upon by fish, and eventually, end up infecting cetaceans (Ahmadpour et al., 2022). Infections caused by *T. gondii* in cetaceans have been linked to a range of diseases, such as neurological disorders, pneumonia, nephritis, and myocarditis, and can lead to severe illness and death (Ahmadpour et al., 2022). *H. nana* can cause hymenolepiasis or dwarf tapeworm infection, particularly in humans, which is typically asymptomatic, but this condition can lead to abdominal pain, diarrhoea, nausea, weakness, and a decrease in appetite when infections are severe (Coello Peralta et al., 2023). However, there are no previous reports of *H. nana* infecting marine animals and as such, the identification of this cestode would require molecular verification for further analysis and discussion. If the species identification is later confirmed, this would be the first record of *H. nana* in odontocetes in South African waters.

### 4.3 Coastal vs. offshore host species

Contrary to our expectation that coastal odontocetes would have higher parasitic prevalence than offshore species due to their proximity to pollutants and runoff from land, this study indicated that the opposite was true: the offshore, deep-diving odontocetes, namely, *S. coeruleoalba*; *S. attenuata*; *G. griseus*; *K. breviceps* and *K. sima,* had a higher prevalence of parasites than those with a more coastal distribution, like *S. plumbea*, *T. aduncus* and *D. delphis* (Table 4).

Previous research has indicated that coastal dolphins tend to have higher parasite loads than offshore marine mammals; with factors such as environmental pollution, sewage and run-off and global climate change contributing to the higher parasite densities found in coastal dolphins (Lafferty and Holt, 2003; de Wet, 2013; Lane et al., 2014). In addition, a study by Gui et al. (2018) described a significant positive correlation between parasitic diseases and high levels of dichlorodiphenyltrichloroethane (DDT), a persistent organic pollutant (POP) previously widely used as insecticide, present in cetaceans, despite not being able to determine the direction of causality. The cetaceans along the southern African coastline were found to have very high levels of DDT, with the levels in *S. plumbea* and *T. aduncus* being among the highest reported for delphinids globally (Gui et al., 2016). As such, we expected these coastal dolphins (some of which were from the same sampling area as those in Gui et al., 2016) to have higher parasitic loads due to a combination of the previous research on cetaceans and possibly compromised immune systems (as a result of pollution).

Offshore dolphins tend to have larger ecological ranges and subsequently feed on a wider range of prey species, compared to coastal dolphins that are limited to narrow bands along the coast, feeding on specific types of prey (Oudejans et al., 2015; Félix and Castro, 2023). These two ecotypes also usually differ in their social behaviour, with coastal dolphins forming more family groups of 12 or fewer individuals, while offshore dolphins usually form larger groups with hundreds of individuals (Oudejans et al., 2015; Félix and Castro, 2023). Offshore dolphins may therefore be exposed to a wider range of parasite species due to their larger ecological and feeding ranges. In addition, their increased social behaviour can result in higher ectoparasite exchange between individuals, due to the larger group sizes. These factors may have contributed to the higher parasitic loads seen in offshore odontocetes in this study.

### 4.4 Prevalence and presence of parasitic infection: differences by sex, age class, collection method and temporal trend

The eight odontocete species investigated displayed various degrees of infection, with parasites infecting both sexes and all age classes (Table 5).

Most male host species had higher parasitic prevalences when compared to female host species (Table 5). There is very little information on how the host's sex affects the incidence of parasites in cetaceans, but no significant differences in parasitic loads between the sexes has been reported previously (Mattiucci and Nascetti, 2006; García-Gallego et al., 2023). Similarly, our study found no significant differences in the presence and absence of parasites between the sexes in all eight odontocete species (Table 6).

There is no clear pattern in the level of parasitic prevalence among the age classes of different odontocete species (Table 5). Model results showed that age class is significantly associated with parasite presence of only the stranded individuals of two species, *T. aduncus* and *S. coeruleoalba,* where the probability of parasite presence is significantly lower in neonates/calves (Table 6). In general, larger or older individuals tend to have more parasites compared to younger or smaller individuals, because they are exposed to infection over longer periods of time (Poulin, 1995; Alwis et al., 2024) and, in the case of trophically transmitted parasites, have a wider dietary range (Raga et al., 2009). It is also known that certain parasites, such as *Halocercus pingi*, can infect calves through maternal transfer via the placenta or the mother's milk (Parsons et al., 2001; Fauquier et al., 2009). The immune system in calves is not fully developed, which leaves them somewhat more susceptible to parasite infections and their effects (Parsons et al., 2001). However, the relationship between parasite infection and host body size is complex and dependent on several parameters, such as the parasite and host type, as well as the host's age, sex, diet and mobility (Poulin, 1995; Alwis et al., 2024).

The observed variations in parasite prevalence over time could be caused by a number of factors, including changes in diet or water temperatures (Geraci and Aubin, 1987; Van Bressem et al., 2009). Although parasitic prevalence appeared to be lower in the 1990–1999 for bycaught individuals and lower in the 1970–1979 for stranded individuals (Table 5), which seems to agree with the model results (Table 6), determining causality and effect between climate change and diet with changes in parasite prevalence is often challenging due to the absence of useful baseline data (Harvell et al., 2002). Parasite prevalence was highest in the most recent years (2000–2015) for some odontocete species, including *S. plumbea* (bycaught: 24.56 %), *T. aduncus* (bycaught: 12.24 % and stranded: 14.10 %), *S. coeruleoalba* (bycaught: 40.0 % and stranded: 31.58 %) and *K. sima* (40.0 %). The models for these odontocete species showed a significantly higher presence of parasites during the same time interval, with the exception of *S. plumbea*, which showed no significant differences between time intervals (Table 6). However, the observed prevalence estimates are of particular importance for *S. plumbea*, as, in addition to a shift in diet and decrease in body condition during the same time interval (Plön et al., *in prep*.), the increasing parasite prevalence could lead to further decline in the health and therefore the population numbers of this already endangered dolphin species.

Out of the three odontocete species for which the probability of collection method playing a role could be assessed, it was only significant for *D. delphis*, with results of the presence/absence models indicating that stranded individuals had a higher probability of parasite presence (Table 6). This result corroborates the observed parasitic prevalence being higher in the stranded individuals of *D. delphis* and *S. coeruleoalba* compared to the bycaught individuals. For *T. aduncus,* parasitic prevalence estimates were similar between the bycaught and stranded individuals (Table 4). These results were expected as stranded dolphins are often sick and more susceptible to parasitic infection, while bycaught individuals are believed to be healthy individuals’ representative of the wild population (Plön et al., 2015).

### 4.5 Conclusion

Parasites can significantly impact the health of marine mammals and can be indicators of health at the individual, population, and environmental levels. Understanding disease epidemiology, determining the health state of cetacean populations, and creating efficient conservation and management plans all depend on the precise morphological identification of the parasites impacting these marine animals. Although the preservation methods for this collection of parasites were not optimal for staining or genetic analyses, the resource remains extremely valuable and rare. Where possible, future studies should incorporate molecular techniques to achieve species-level parasite identification; however, the absence of such methods should not deter researchers from drawing meaningful insights from datasets like this. The present study is the first comprehensive investigation of parasites infecting small cetaceans in the southern African subregion and thus presents an important baseline for further investigations. Thus, we hope that this study could serve as baseline data for future parasite studies in cetaceans off the South African coastline.

## CRediT authorship contribution statement

**Inge A. Adams:** Writing – review & editing, Writing – original draft, Methodology, Investigation, Formal analysis. **Natasha Roussouw:** Writing – review & editing, Writing – original draft, Methodology, Investigation, Formal analysis. **Cecile Reed:** Writing – review & editing, Supervision, Resources, Conceptualization. **Gin Swen Ham:** Formal analysis, Methodology, Writing – review & editing. **Stephanie Plön:** Writing – review & editing, Writing – original draft, Visualization, Supervision, Resources, Project administration, Methodology, Investigation, Funding acquisition, Conceptualization.

## Funding Sources

This work was supported by a National Research Foundation (NRF) of South Africa ‘Collaborative Postgraduate Training Programme at Higher Education Institutions (HEI) in partnership with other universities, industry and government’ grant to SP (grant id: 92925), sponsorship agreements with the International Fund for Animal Welfare (IFAW) and Exxon Mobil to SP, a Kate Sanderson bequest grant (grant id: 2021A-006) from the International Union for the Conservation of Nature (IUCN) to SP, and co-funding from BioConsult SH Research and Conservation gGmbH, Germany.

## Conflicts of interest

The authors declare that there are no competing interests.
