# Supplementary material for: Long-term host parasite dynamics in eight odontocete species from south-eastern South Africa
Source: Int J Parasitol Parasites Wildl. 2025 Sep 5;28:101133. doi: 10.1016/j.ijppaw.2025.101133 (PMC12481713; doi:10.1016/j.ijppaw.2025.101133)
Supplement: Multimedia component 1 [file mmc1.docx]

**Supplementary Material 1**

Summary of Firth’s logistic regression models testing the presence and absence of parasites across eight odontocete species in southern African waters. Each model assesses the effect of age class (adult, juvenile/subadult and neonate/calf), sex, temporal trend (1970–1979, 1980–1989, 1990–1999 and 2000–2015) and if applicable, collection method (bycatch or stranding). Values shown are coefficient estimates (log-odds scale), standard errors, 95% confidence intervals and *p*-values. Significance is denoted by asterisks: *p*<0.05 (*), *p*<0.01 (**), *p*<0.001 (***).

| **Term** | **Estimate** | **SE** | **Lower 95% CI** | **Upper 95% CI** | **P-value** |
| --- | --- | --- | --- | --- | --- |
| **Full model: *Delphinus delphis*** | | | | | |
| **(Intercept)** | -3.648 | 0.894 | -5.935 | -2.11 | **0***** |
| **Juvenile/subadult** | 0.113 | 0.48 | -0.956 | 1.013 | 0.821 |
| **Neonate/calf** | -0.129 | 0.443 | -1.09 | 0.725 | 0.777 |
| **Male** | -0.066 | 0.336 | -0.755 | 0.615 | 0.85 |
| **1980-1989** | 0.907 | 0.881 | -0.609 | 3.173 | 0.27 |
| **1990-1999** | -1.309 | 1.047 | -3.407 | 1.142 | 0.256 |
| **2000-2015** | 0.474 | 0.879 | -1.044 | 2.736 | 0.58 |
| **Stranding** | 1.294 | 0.404 | 0.449 | 2.102 | **0.003**** |
| **Bycatch-only model: *Delphinus delphis*** | | | | | |
| **(Intercept)** | -3.36 | 1.409 | -8.227 | -1.275 | **0***** |
| **Juvenile/subadult** | 0.045 | 0.526 | -1.149 | 1.03 | 0.934 |
| **Neonate/calf** | -0.454 | 0.657 | -2.084 | 0.732 | 0.487 |
| **Male** | 0.186 | 0.391 | -0.625 | 0.99 | 0.649 |
| **1980-1989** | 0.486 | 1.416 | -1.632 | 5.358 | 0.722 |
| **1990-1999** | -1.392 | 1.525 | -3.98 | 3.558 | 0.447 |
| **2000-2015** | 0.283 | 1.439 | -1.922 | 5.172 | 0.843 |
| **Stranding-only model: *Delphinus delphis*** | | | | | |
| **(Intercept)** | -1.887 | 0.977 | -4.31 | -0.097 | **0.038*** |
| **Juvenile/subadult** | 0.675 | 0.969 | -1.723 | 2.573 | 0.528 |
| **Neonate/calf** | 0.173 | 0.628 | -1.154 | 1.495 | 0.794 |
| **Male** | -0.754 | 0.599 | -2.055 | 0.491 | 0.233 |
| **1980-1989** | 1.143 | 0.969 | -0.65 | 3.534 | 0.223 |
| **1990-1999** | -1.881 | 1.564 | -6.892 | 1.113 | 0.217 |
| **2000-2015** | 0.041 | 0.941 | -1.722 | 2.392 | 0.967 |
| **Full model: *Tursiops aduncus*** | | | | | |
| **(Intercept)** | -4.121 | 0.851 | -6.337 | -2.729 | **0***** |
| **Juvenile/subadult** | 0.244 | 0.291 | -0.334 | 0.821 | 0.407 |
| **Neonate/calf** | -0.078 | 0.27 | -0.616 | 0.458 | 0.774 |
| **Male** | 0.227 | 0.237 | -0.243 | 0.701 | 0.343 |
| **1980-1989** | 1.959 | 0.839 | 0.593 | 4.161 | **0.002**** |
| **1990-1999** | 0.192 | 0.898 | -1.362 | 2.458 | 0.83 |
| **2000-2015** | 2.029 | 0.835 | 0.678 | 4.226 | **0.001**** |
| **Stranding** | 0.292 | 0.269 | -0.264 | 0.811 | 0.294 |
| **Bycatch-only model: *Tursiops aduncus*** | | | | | |
| **(Intercept)** | -4.664 | 1.432 | -9.521 | -2.636 | **0***** |
| **Juvenile/subadult** | 0.532 | 0.344 | -0.149 | 1.226 | 0.126 |
| **Neonate/calf** | 0.322 | 0.318 | -0.306 | 0.963 | 0.316 |
| **Male** | 0.141 | 0.273 | -0.404 | 0.687 | 0.612 |
| **1980-1989** | 2.39 | 1.424 | 0.385 | 7.24 | **0.012*** |
| **1990-1999** | 0.297 | 1.488 | -1.976 | 5.192 | 0.837 |
| **2000-2015** | 2.377 | 1.423 | 0.376 | 7.227 | **0.013*** |
| **Stranding-only model: *Tursiops aduncus*** | | | | | |
| **(Intercept)** | -2.532 | 0.872 | -4.794 | -1.048 | **0***** |
| **Juvenile/subadult** | -0.43 | 0.587 | -1.718 | 0.703 | 0.469 |
| **Neonate/calf** | -1.36 | 0.567 | -2.623 | -0.266 | **0.014*** |
| **Male** | 0.245 | 0.469 | -0.702 | 1.232 | 0.614 |
| **1980-1989** | 0.744 | 0.931 | -0.958 | 3.067 | 0.413 |
| **1990-1999** | 0.278 | 0.974 | -1.589 | 2.649 | 0.779 |
| **2000-2015** | 1.395 | 0.875 | -0.124 | 3.655 | 0.075 |
| **Full model: *Stenella coeruleoalba*** | | | | | |
| **(Intercept)** | -2.396 | 1.042 | -4.93 | -0.501 | **0.011*** |
| **Juvenile/subadult** | -0.125 | 0.699 | -1.662 | 1.246 | 0.862 |
| **Neonate/calf** | -0.945 | 0.747 | -2.691 | 0.465 | 0.198 |
| **Male** | -0.487 | 0.536 | -1.606 | 0.601 | 0.379 |
| **1980-1989** | 1.286 | 0.943 | -0.454 | 3.633 | 0.155 |
| **1990-1999** | 1.435 | 0.993 | -0.441 | 3.847 | 0.138 |
| **2000-2015** | 2.08 | 0.925 | 0.422 | 4.403 | **0.012*** |
| **Stranding** | 0.315 | 0.645 | -0.94 | 1.778 | 0.635 |
| **Bycatch-only model: *Stenella coeruleoalba*** | | | | | |
| **(Intercept)** | -1.608 | 1.681 | -7.006 | 2.168 | 0.381 |
| **Juvenile/subadult** | -0.076 | 1.575 | -5.2 | 3.867 | 0.967 |
| **Neonate/calf** | 0.866 | 1.172 | -1.588 | 3.727 | 0.489 |
| **Male** | 0.089 | 1.036 | -2.335 | 2.685 | 0.939 |
| **1980-1989** | 0.323 | 1.658 | -3.73 | 5.566 | 0.861 |
| **1990-1999** | -1.37 | 2.084 | -7.285 | 4.52 | 0.592 |
| **2000-2015** | 0.754 | 1.84 | -3.277 | 6.428 | 0.711 |
| **Stranding-only model: *Stenella coeruleoalba*** | | | | | |
| **(Intercept)** | -2.07 | 0.924 | -4.401 | -0.457 | **0.009**** |
| **Juvenile/subadult** | -0.139 | 0.731 | -1.707 | 1.293 | 0.851 |
| **Neonate/calf** | -2.581 | 1.441 | -7.496 | -0.307 | **0.022*** |
| **Male** | -0.333 | 0.613 | -1.598 | 0.93 | 0.601 |
| **1980-1989** | 1.326 | 0.987 | -0.515 | 3.716 | 0.163 |
| **1990-1999** | 2.01 | 1.057 | 0.022 | 4.508 | **0.047*** |
| **2000-2015** | 1.832 | 0.968 | 0.076 | 4.2 | **0.04*** |
| **Bycatch-only model: *Sousa plumbea*** | | | | | |
| **(Intercept)** | -1.498 | 1.654 | -6.525 | 1.3 | 0.319 |
| **Juvenile/subadult** | 0.168 | 0.522 | -0.907 | 1.242 | 0.756 |
| **Neonate/calf** | 0.253 | 0.788 | -1.534 | 1.746 | 0.758 |
| **Male** | -0.184 | 0.501 | -1.198 | 0.857 | 0.723 |
| **1980-1989** | -0.667 | 1.614 | -3.382 | 4.318 | 0.7 |
| **1990-1999** | -3.048 | 2.07 | -8.389 | 2.279 | 0.197 |
| **2000-2015** | 0.4 | 1.586 | -2.231 | 5.363 | 0.795 |
| **Stranding-only model: *Grampus griseus*** | | | | | |
| **(Intercept)** | -2.759 | 1.749 | -7.893 | 0.299 | 0.08 |
| **Juvenile/subadult** | 1.15 | 0.869 | -0.575 | 3.089 | 0.192 |
| **Neonate/calf** | -1.15 | 1.143 | -3.873 | 1.232 | 0.344 |
| **Male** | 0.563 | 0.764 | -1.045 | 2.303 | 0.494 |
| **1980-1989** | 0.202 | 1.71 | -2.873 | 5.289 | 0.907 |
| **1990-1999** | 1.037 | 1.757 | -2.129 | 6.177 | 0.546 |
| **2000-2015** | -0.864 | 2.158 | -6.325 | 4.564 | 0.699 |
| **Stranding-only model: *Kogia breviceps*** | | | | | |
| **(Intercept)** | -3.615 | 1.543 | -9.122 | -1.133 | **0.001***** |
| **Juvenile/subadult** | 0.023 | 1.351 | -2.936 | 3.169 | 0.987 |
| **Neonate/calf** | -2.87 | 1.707 | -8.454 | 0.265 | 0.077 |
| **Male** | 1.403 | 1.281 | -1.193 | 6.151 | 0.304 |
| **1980-1989** | 3.697 | 1.662 | 0.678 | 9.12 | **0.014*** |
| **1990-1999** | 3.671 | 1.717 | 0.561 | 9.352 | **0.018*** |
| **2000-2015** | 0.641 | 2.01 | -4.721 | 6.001 | 0.766 |
| **Stranding-only model: *Kogia sima*** | | | | | |
| **(Intercept)** | -3.139 | 1.004 | -5.862 | -1.413 | **0***** |
| **Juvenile/subadult** | 1.381 | 0.993 | -0.603 | 3.827 | 0.171 |
| **Neonate/calf** | -0.091 | 1.069 | -2.599 | 2.006 | 0.934 |
| **Male** | -0.823 | 1.025 | -3.499 | 1.155 | 0.433 |
| **1980-1989** | 2.626 | 1.125 | 0.489 | 5.532 | **0.015*** |
| **1990-1999** | 3.452 | 1.712 | 0.117 | 7.886 | **0.043*** |
| **2000-2015** | 3.402 | 1.376 | 0.963 | 7.433 | **0.004**** |
| **Stranding-only model: *Stenella attenuata*** | | | | | |
| **(Intercept)** | -1.099 | 1.633 | -6.082 | 1.832 | 0.469 |
| **Juvenile/subadult** | 0.78 | 2.011 | -4.557 | 6.126 | 0.715 |
| **Neonate/calf** | -0.804 | 1.537 | -5.824 | 1.934 | 0.603 |
| **Male** | 0.631 | 1.039 | -1.572 | 3.241 | 0.575 |
| **1980-1989** | -1.589 | 2.245 | -7.245 | 3.959 | 0.504 |
| **1990-1999** | 0.029 | 1.841 | -3.49 | 5.202 | 0.988 |
| **2000-2015** | -1.099 | 1.633 | -6.082 | 1.832 | 0.469 |
